# Metabolic Assessment of Human Induced Pluripotent Stem Cells-Derived Astrocytes and Fetal Primary Astrocytes: Lactate and Glucose Turnover

**DOI:** 10.3390/bios12100839

**Published:** 2022-10-08

**Authors:** Isabelle Matthiesen, Rohollah Nasiri, Alessandra Tamashiro Orrego, Thomas E. Winkler, Anna Herland

**Affiliations:** 1 Division of Micro and Nanosystems, Department of Intelligent Systems, School of Electrical Engineering and Computer Science, KTH Royal Institute of Technology, 10044 Stockholm, Sweden; 2 CVRM Safety, Clinical Pharmacology and Safety Sciences, R&D, AstraZeneca, 43150 Gothenburg, Sweden; 3 Division of Nanobiotechnology, Department of Protein Science, Science for Life Laboratory, KTH Royal Institute of Technology, 17165 Solna, Sweden; 4 AIMES, Center for the Advancement of Integrated Medical and Engineering Sciences, Department of Neuroscience, Karolinska Institute, 17177 Solna, Sweden; 5 Institute of Microtechnology & Center of Pharmaceutical Engineering, Technische Universität Braunschweig, 38106 Braunschweig, Germany

**Keywords:** astrocytes, brain metabolism, human iPSC-derived astrocytes (hiAstrocytes), human fetal astrocytes (HFAs), glucose, lactate, biosensor

## Abstract

Astrocytes represent one of the main cell types in the brain and play a crucial role in brain functions, including supplying the energy demand for neurons. Moreover, they are important regulators of metabolite levels. Glucose uptake and lactate production are some of the main observable metabolic actions of astrocytes. To gain insight into these processes, it is essential to establish scalable and functional sources for in vitro studies of astrocytes. In this study, we compared the metabolic turnover of glucose and lactate in astrocytes derived from human induced pluripotent stem cell (hiPSC)-derived Astrocytes (hiAstrocytes) as a scalable astrocyte source to human fetal astrocytes (HFAs). Using a user-friendly, commercial flow-based biosensor, we could verify that hiAstrocytes are as glycogenic as their fetal counterparts, but their normalized metabolic turnover is lower. Specifically, under identical culture conditions in a defined media, HFAs have 2.3 times higher levels of lactate production compared to hiAstrocytes. In terms of glucose, HFAs have 2.1 times higher consumption levels than hiAstrocytes at 24 h. Still, as we describe their glycogenic phenotype, our study demonstrates the use of hiAstrocytes and flow-based biosensors for metabolic studies of astrocyte function.

## 1. Introduction

The human brain consumes about 20% of the body’s energy intake [1]. The major cellular participants for this are the highly interconnected astrocytes and neuronal cells. Astrocytes play multiple vital roles in maintaining and regulating synaptic physiology, neuronal communication, and energy metabolism [2,3]. They are the most glycolytic cells in the brain and supply metabolites to support neurons, the most energy-demanding cells in the central nervous system (CNS) [4]. Moreover, astrocytes are one of the main secretory cells in the body, releasing a range of factors, including neurotransmitters, metabolites, hormones, growth factors and peptides [5]. The brain’s primary energy source is glucose, and astrocytes take up glucose and either store it as glycogen or metabolize it to pyruvate (glycolysis). Pyruvate is converted to lactate, which is then shuttled to neurons, which can back-convert lactate to pyruvate for use in the tricarboxylic acid (TCA) cycle. However, to a lesser extent, neurons can also directly utilize glucose and even release lactate themselves [6]. Therefore, astrocytes are important regulators of metabolite levels, including glucose uptake and lactate production [7].

Using in vivo experiments to define the roles of astrocytes in human brain metabolism process is technically difficult. To understand the metabolic interactions in the human brain, we need detailed in vitro studies. Animal models or animal-derived cells of the CNS do not fully capture human physiology and pathology. Human primary CNS cells are rare and typically fetal, yielding ethical questions and therefore often avoided in industrial applications. As an alternative cell source, cell lines or hiPSC-derived cells can be used to generate functional astrocytes [8]. The biological relevance of each in vitro model, primary, cell line or stem-cell-derived cells must, however, be validated, as we recently showed with two studies using hiPSC-derived astrocytes [9,10].

To investigate the capacity to use hiPSC-derived astrocytes in the context of human brain metabolism, we need to compare them to the most in vivo-like cell source, i.e., human fetal astrocytes (HFAs). HFAs, as their name implies, are obtained and purified from human fetal samples and can be expanded in vitro for a limited number of passages. The human-induced astrocytes (hiAstrocytes) we use are differentiated from a stable neural stem cell state derived from hiPSC [10]. hiAstrocytes show astrocyte marker expression, functional glutamate transporters, neuroinflammatory response, and glutathione secretion. However, whether or not their metabolic actions are comparable has not yet been investigated. Studying the difference in metabolic parameters of these cells can provide insight regarding their applicability for in vitro studies, including disease modeling and drug screening. 

**Figure 1:**
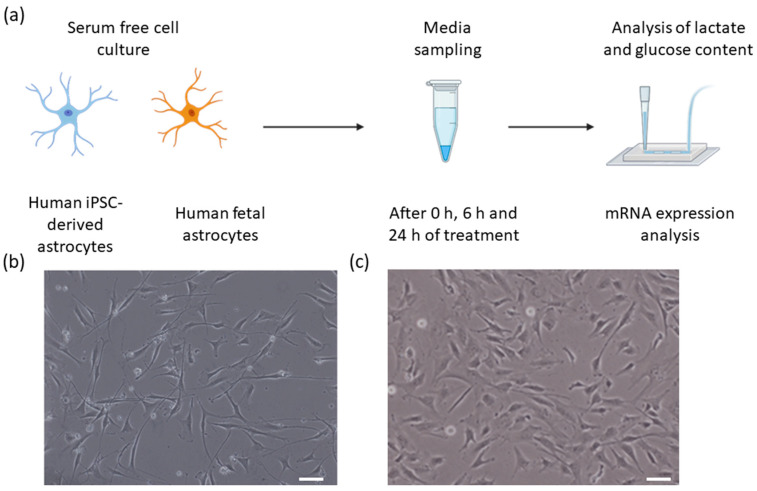
(**a**) An experimental overview of this study illustrating the two cell types, media sampling timepoints, flow sensor analysis of lactate and glucose, and mRNA expression analysis. (**b**,**c**) Bright-field images of (**b**) Human iPSC-derived astrocytes, and (**c**) human primary astrocytes. Scale bars 50 µm.

To our knowledge, there is no reported direct comparison between hiAstrocytes and HFAs in the literature so far. In the present study, in vitro monocultures of hiAstrocytes and HFAs are compared for metabolic characteristics (Figure 1a). We carried out a set of experiments where hiAstrocytes and HFAs were cultured in the same defined media with a specific concentration of glucose (5.5 mM) for 24 h. Lactate production and glucose consumption by the cells were then evaluated. Existing biometabolic analysis methodologies, such as colorimetric or fluorescence-based assay kits and UV-resonance Raman spectroscopy, can suffer from high variability and high cost, are indirect measurements, and typically require different reagents and handling steps. As an alternative, biosensors offer direct and real-time detection of many biological and chemical substances with high specificity and sensitivity, small size, and cost-effectiveness [11]. Commercial, multiplexed biosensors are now available on the market and have previously been applied to monitor fermentation or skin explants [12,13]. Using biosensors offers clear advantages over other existing biometabolic analysis approaches, supporting the choice of an enzymatic biosensor for our metabolic study in this paper.

Here, we demonstrate how a commercial, multiplexed biosensor for both glucose and lactate can be applied in detailed metabolic studies. Additionally, we performed a smaller transcriptomic analysis to compare the expression of a few major metabolic enzymes and transporters in the two astrocytic cell types. Figure 1a shows the overview of the cells, sampling process, and analysis steps.

## 2. Materials and Methods

### 2.1. Cell Models/Source and Cell Culture

hiPSCs were differentiated to astrocytes based on our recent protocol [10]. Fetal human cortical astrocytes were obtained from ScienCell (#1800). Cells were cultured according to suppliers’ instructions and used at passage <8 post-thawing. One million cells were thawed in complete astrocyte media (AM; ScienCell 1801). For assays, 500,000 cells/well were seeded in 6-well cell culture plates coated with 1x attachment factor (AF; Thermo S-006-100). On the next day of subculture (day 1), the media were changed to a 1:1 mixture of AM and “defined media,” and on day 2 to defined media only. Our defined media were composed of DMEM without glucose and glutamine (Thermo A14430-01) supplemented with 1% G-5 (Thermo 17503012; serum-free supplement for growth and expression of glial cells), 1% Penicillin-Streptomycin (P/S; ScienCell 0503), 10mM GlutaMax (Sigma 35050-38), 25 mM HEPES (EMD 391340), and 5.55 mM glucose (Sigma G7528) to match the AM glucose content. We used these defined media to have better control over the initial concentration of glucose within the media. Exemplary bright-field images of the cells on day 2 are shown in Figure 1b,c.

### 2.2. Sample Collection

At 0 h, 6 h, and 24 h of the cultivation phase with defined media (day 3), 100 µL of media was sampled into a 96-well collection plate (Axygen P-96-450V-C). At 24 h, the cells were washed with PBS and then were lysed with 350 µL RLT buffer/well (Qiagen 1015750). The media samples and lysates were stored at −80 °C until further analysis.

### 2.3. mRNA and dsDNA Quantification

We followed standard qPCR methods [10] to assess mRNA expression levels for the hiAstrocytes and HFA after 24 h of cultivation with defined media. Specifically, key metabolic enzyme markers including 3-hydroxybuturate dehydrogenase 2 (BDH2), 4-aminobutyrate aminotransferase (ABAT), 3-hydroxymethyl-3-methylglutaryl-CoA lyase (HMGCL), 3-oxoacid CoA-transferase 1 (OXCT1), transporter markers solute carrier family 6 member 1 (SLC6A1) and solute carrier family 16 member 1 (SLC16A1) were measured. Replicates with Ct > 35, Ct < 10, or where duplicate measurements of the same sample varied by more than 1 Ct, were excluded from analysis.

Additionally, to serve as normalization for cell numbers regarding the metabolic parameters between cell types and individual samples, dsDNA content was measured using a Quant iT Pico Green kit (Thermo P11496) and a fluorescent plate reader.

### 2.4. Lactate Assay

As an internal process control, we employed a commercial kit to assay lactate (Sigma-Aldrich MAK064) in the collected media samples. Measurements were made according to manufacturer protocols using a plate reader for fluorescent readout. Media samples were diluted 50 times to conform to assay requirements in terms of linear range.

### 2.5. Biosensor Metabolic Readout

**Figure 2:**
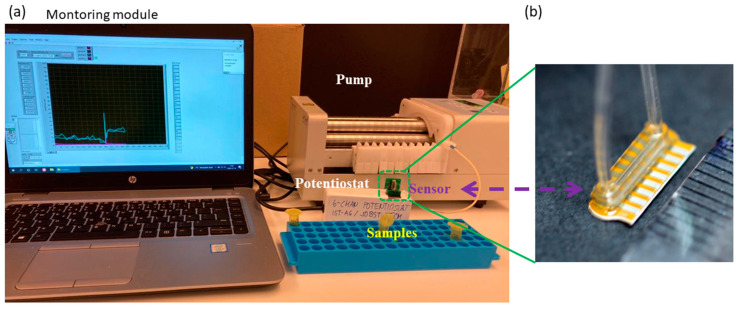
(**a**) Experimental setup for detecting glucose and lactate in the samples using the flow-through biosensor. (**b**) Image of the utilized B.LV5 flow-through sensor for lactate and glucose measurement in this study. A ruler on the lower right shows millimeter markings demonstrating the size of the sensor, i.e., with the flow cell it is just under one cm.

To measure the consumption of glucose and the production of lactate by astrocytes, a commercial flow-through enzymatic amperometric biosensor (Jobst Technologies B.LV5) was utilized. This sensor features four enzyme-modified electrodes—two each for lactate and glucose detection—as well as two unmodified electrodes for baseline subtraction. The calibrated sensitivity of the utilized biosensor for glucose and lactate are 0.7 nA/mM and 0.9 nA/mM, respectively. Using calibration samples, we confirmed that this was valid in both buffer solutions as well as our defined media. The manufacturer specifies a linear range of at least 0.05–25 mM for glucose and 0.02–15 mM for lactate (though based on our experience this is narrower in cell culture media). The sensor’s microfluidic packaging enables measurements with less than 10 µL sample volume. The collected samples were perfused through the biosensor using a peristaltic pump (Lachat RP-150; PharMed BPT tubing) and read out by connecting the biosensor to a potentiostat (Jobst Technologies SIX) and a custom monitoring UI (National Instruments LabVIEW) (Figure 2). The operating flow rate in our measurements was ~1.3 µL/min. We followed manufacturer recommendations for sensor cleaning and maintenance.

### 2.6. Statistical Analysis

**Figure 3:**
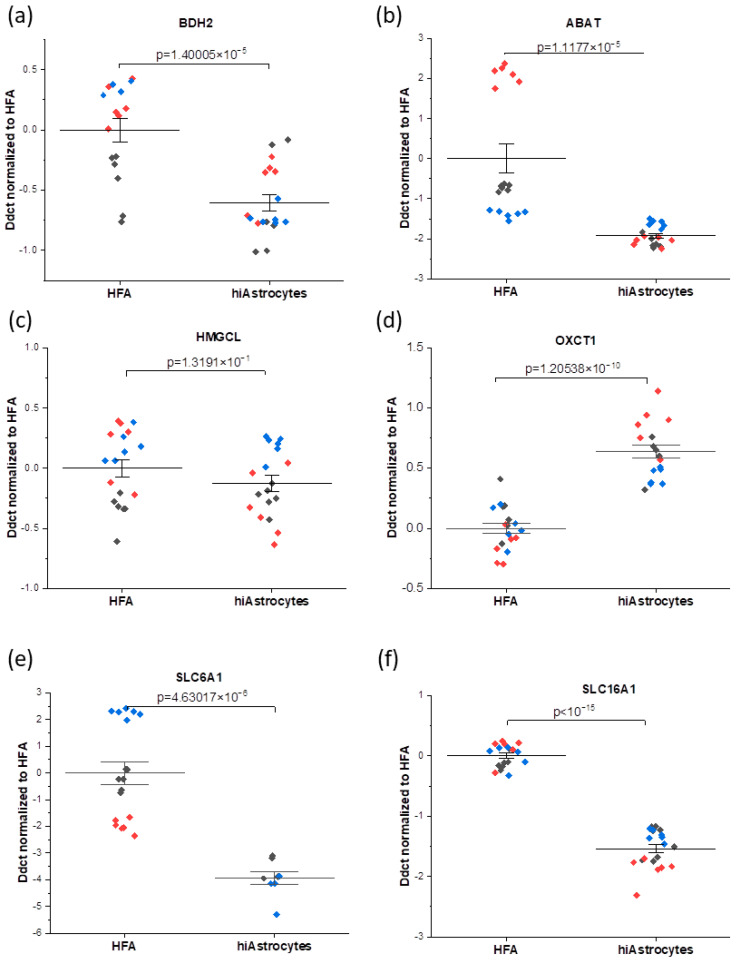
mRNA analysis profiles showing the comparison between the HFA and hiAstrocytes for the genes connected to metabolic enzymes (**a**–**d**): (**a**) BDH2 (**b**) ABAT, (**c**) HMGCL, (**d**) OXCT1, and transporters (**e**,**f**): (**e**) SLC6A1 (**f**) SLC16A1. (i.e., the data here are normalized to HFA control). The data were collected as technical duplicates, with distinct colors indicating different experimental rounds. Whiskers indicate ± SE around the bar that represents the mean. Genes of interest are normalized to the housekeeping gene GAPDH.

Samples were collected in triplicates from three individual experiments (N = 3 independent biological replicates; thus, n = 3 × 3 = 9 samples total). Due to an outlier value in dsDNA quantification for one of the experimental rounds (in disagreement with visual observations, thus likely due to incomplete lysis), only data from N = 2 (i.e., total n = 6 wells) are used where dsDNA normalization is required. Specifically, we normalize metabolic data by the relative dsDNA content in each well and assume an average 100% cell survival across all wells/conditions in the metabolic rate calculations. Based on microscopy observations, this is a conservative assumption (i.e., potentially overestimating cell survival), thus providing us conservative metabolic rates (i.e., values are at least lower limits). All statistical analyses were carried out in Origin Pro (Origin Lab, Northampton, MA, USA), and *p*-values were derived with linear mixed models (LMM). For the qPCR data in Figure 3, any technical duplicates with a difference of more than 1 Ct were excluded.

## 3. Results and Discussion

### 3.1. mRNA Analysis

We studied the mRNA expression of a set of genes that have been connected to metabolic activities (BDH2, ABAT, HMGCL, OXCT1, SLC6A1, and SLC16A1), which are described in Table 1.

It is worth mentioning that in our recent work [10], we have studied the mRNA expression of the astrocyte-specific markers, including glial fibrillary acidic protein (GFAP), S100 calcium-binding protein B (S100B), and cluster of differentiation 44 (CD44) for both HFAs and hiAstrocytes. When comparing HFAs and hiAstrocytes in terms of astrocyte-specific marker expression, we observed that both cell types have statistically equivalent levels of expression for GFAP and CD44, however hiAstrocytes showed ~5 DDCt increased expression of S100B [10].

Overall, here we interestingly observe higher consistency of mRNA expression for the hiAstrocytes, with a smaller median absolute deviation of DDCt values—which also varies less between genes of interest—than the HFA (0.19 ± 0.06 versus 0.54 ± 0.63, respectively). For genes that have been connected to metabolic enzymes (BDH2, ABAT, HMGCL, and OXCT1), it can be observed that all considered genes have biologically equivalent levels of expression, i.e., within 1 DDct for hiAstrocytes and HFA (disregarding, for HFA, the outlying ABAT results in one round). On the other hand, the transporter markers (SLC6A1, SLC16A1) are the genes that have biologically significantly lower expression in the hiAstrocytes (Figure 3). The GABA transporter SLC6A1 expression in these cells is not directly linked to glucose turnover but to GABA uptake. SLC16A1 also has a roughly three-fold lower expression in hiAstrocytes, though the difference with HFA is less pronounced than for SLC6A1. SLC16A1, which encodes for the monocarboxylate transporter 1, is one of the major transporters of lactate.

We additionally note that the qPCR house-keeping gene GAPDH, encoding a protein in the glycolysis pathway, showed the same expression levels in hiAstrocytes compared to HFAs for the same amount of assayed mRNA (Figure S1).

In summary, the mRNA expression of intracellular metabolic enzymes that we studied are on the same level (largely within 1 DDCt) in hiAstrocytes compared to HFAs, suggesting a comparable inherent metabolic capacity. However, the difference in important transporters for metabolic substrates could imply that the hiAstrocytes cannot carry out the same metabolic processes as HFAs. These processes can indeed be very context-specific and fast-regulated in different media, as we have verified higher and more functional glutamate uptake in hiAstrocytes compared to HFAs in another study [10]. Finally, the higher consistency in mRNA expression of hiAstrocytes highlights one of the key potential advantages of this type of cell source compared to primary cells.

Future works with optimized protocols can provide more relevant gene expression values for hiAstrocytes. However, our data also confirm the capacity of hiAstrocytes for metabolic studies, which can be used as a scalable cell source for CNS-related research including disease modeling and drug screening, as well as precision medicine by using patient’s specific cells.

### 3.2. Glucose Consumption and Lactate Production

**Figure 4:**
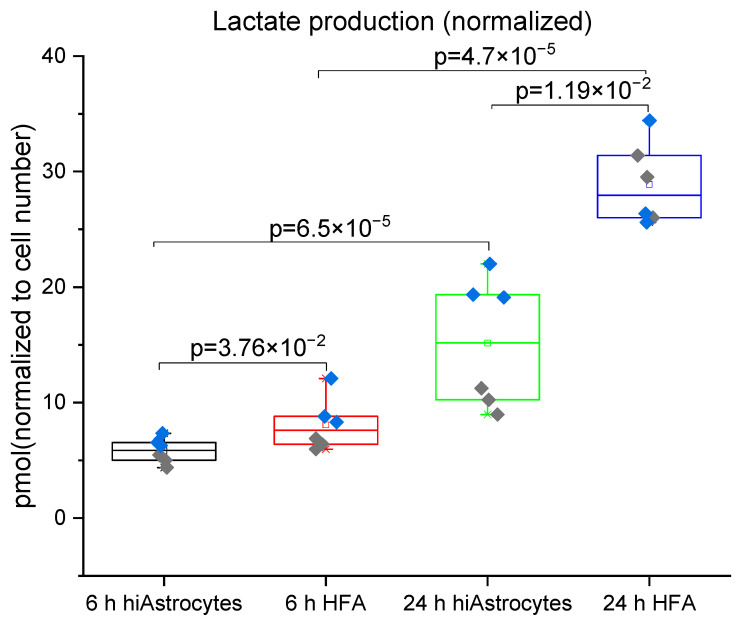
Lactate production for hiAstrocytes and HFA using a flow-based biosensor: Lactate production of hiAstrocytes and HFA after 6 h and 24 h treatment with defined media. Data were normalized to estimated cell numbers and relative dsDNA content of the samples at 24 h. Data are presented both as a box indicating the 25th–75th percentile, including a median line with 95% CI for the whiskers, with the addition of individual data points where colors represent independent experimental rounds. Corresponding *p*-values are presented in the graph, with a significance cutoff of 1 × 10^−2^ to correct for multiple comparisons.

**Figure 5:**
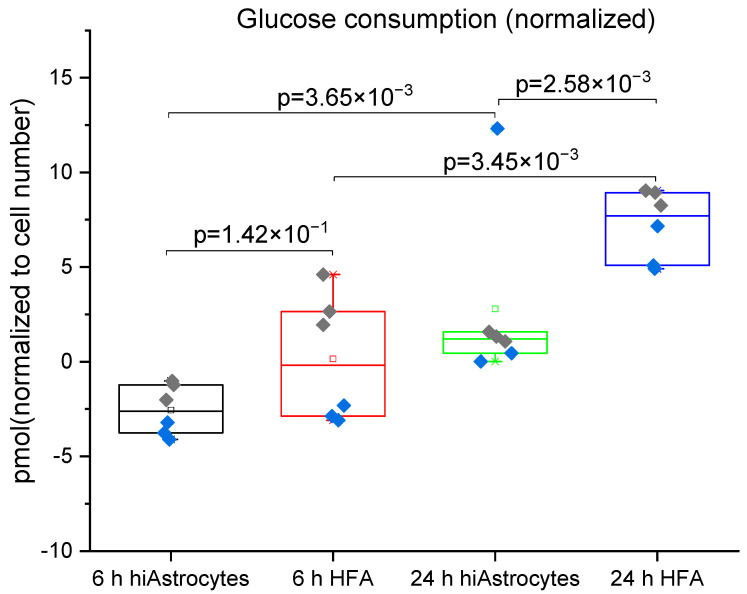
Glucose consumption measured for hiAstrocytes and HFA using a flow-based biosensor: Glucose consumption of hiAstrocytes and HFA after 6 h and 24 h treatment with defined media. Data were normalized to estimated cell numbers and relative DNA content of the samples at 24 h. Data are presented both as a box indicating the 25th–75th percentile, including a median line with 95% CI for the whiskers, with the addition of individual data points where colors represent independent experimental rounds. All corresponding *p*-values are presented in the graph, with a significance cutoff of 1 × 10^−2^ to correct for multiple comparisons. The baseline drift of the sensor proves to be the limiting factor for the high-concentration analyte glucose, evidenced by negative values at 6h.

We next investigated the metabolism of hiAstrocytes and HFA in terms of glucose consumption and lactate production in the defined media over 24 hrs. With the flow-based biosensor, we could simultaneously measure glucose and lactate concentrations C (based on amperometry and sensor calibration). To be able to compare cell types, we normalized the values by the estimated cell numbers #Cells from optical observations and the relative dsDNA content of each well at 24 h (Figure 4 and Figure 5, also accounting for initial glucose/lactate concentrations, and 2 mL media volume). Metabolic rates (Table 2) are then calculated as: $Rate\;=\;\left|\frac{\left(C_{24h}-C_{6h}\right)\;\times \;2ml}{\#Cells\times \left(24h-6h\right)}\right|$. In these serum-free conditions, neither cell type shows any considerable proliferation over the 24 h treatment period (data not shown), suggesting that we can compare normalized metabolites across the different time points. We observe a significant amount of lactate production from the HFAs at as early as 6 h, whereas the hiAstrocytes have a roughly 25% slower lactate production. Both types of astrocytes show a significant increase in lactate production from the 6 h to 24 h time point; however, hiAstrocytes continue to produce less lactate, now by roughly 55%. This may indicate that the hiAstrocytes adapt slower to our defined media and take longer to recover from the media change.

The glucose consumption by the cells shown in Figure 5 also matches these data. In line with the lactate results, between the 6 h and 24 h measurement, both cell types show a significant increase in glucose consumption. HFAs have significantly greater glucose consumption than the hiAstrocytes at the 24 h time point (but not the 6 h time point). The higher variability in the glucose data reflects the lower sensitivity of the sensor for this much higher-concentration analyte. In both cases, baseline drift (rather than detection limit) proves to be a limiting factor, as most clearly evidenced by the negative values present. For lactate production, a standard lactate assay supports the trends observed with the biosensor (Figure S2), but this assay in fact exhibits higher variability than the sensor.

HFAs from the same cell source were indeed previously established to be glycolytic in a similar serum-free environment [4]. The present data are, to our knowledge, the first direct comparison between HFA and hiPSC-derived counterparts. Specifically, we observe that the hiAstrocytes are glycolytic but have approximately 50% lower lactate production and 60% lower glucose consumption than HFAs between 6 h and 24 h (although the sensor baseline drift increases the uncertainty of the glucose estimate).

Table 2 presents a summary of the metabolic rates found in our study together with rates from previous metabolic studies. In a previous study [4] using primary astrocytes, lactate production was measured by mass spectrometry and observed to be lower compared to our findings. Given the defined, minimal composition of our media, it is likely that the high lactate production rate—and high excess lactate/glucose ratio—originates from glutamine breakdown [14]. Glutaminolysis, as a metabolic process, catabolizes glutamine to produce lactate and ATP [15]. High metabolic utilization of glutamine has previously been observed in cortical rat astrocytes [16]. In a broader context, Dong et al. [14], using enzymatic test kits (Roche Diagnostics), demonstrated both lower glucose and lactate values compared to our work, 0.259 and 0.296 pmol/cell/h, respectively, for hepatocytes cultured in vitro. Additionally, in another study by Bavli et al. [17], hepatocytes were cultured in a bioreactor, and using amperometric glucose and lactate sensors (purchased from BioSensor Technology), the metabolic rates for glucose uptake and lactate production were determined to 0.144, and 0.180 pmol/cell/h, respectively, which are also lower values than we measured for astrocytes in this study. This confirms that astrocytes are more energy-demanding cells with a higher glucose consumption rate compared to hepatocytes.

In summary, the glucose metabolism in our study is on par with liver cells for hiAstrocytes, and lactate metabolism is significantly higher than in hepatocytes for both of our astrocyte types. When comparing the lactate/glucose ratios, we observed higher values that could originate from glutamine breakdown (glutaminolysis), which also leads to higher lactate production, as mentioned earlier.

**Table 2:** Comparison of the median metabolic rate of the cells in our study and previous studies.

Analyte	HFA (Our Study)	hiAstrocytes (Our Study)	HFA Ref. [4]	Human Hepatocytes Ref. [18]	Human Hepatocytes Ref. [17]
Glucose consumption rate [pmol/cell/h]	0.398 ± 0.11	0.189 ± 0.05	NA	0.259	0.144
Lactate production rate [pmol/cell/h]	1.177 ± 0.15	0.510 ± 0.23	0.298	0.296	0.180
Lactate/glucose ratio	2.95	2.69	NA	1.14	1.25

As we discussed earlier, astrocytes are the most glycolytic cells in the brain. They are one of the key cells in regulating energy metabolism for the brain, as they provide energy for neurons by producing lactate and ketones. Metabolic assessment of astrocytes can pave the way for better understanding of its metabolic rate in terms of glucose uptake and lactate production rate. Moreover, characterization of the metabolic parameters of astrocytes is still lacking; therefore, this study presents the quantified approach for finding the glucose uptake and lactate production in human astrocytes to get insights on the rate of glucose uptake and lactate production for these cells. Additionally, in this work we studied the hiPSC-derived astrocytes along with primary astrocytes and the capacity of hiPSC-derived astrocytes in brain metabolism compared to HFAs to confirm its usability as a scalable cell source for in vitro metabolic studies for disease modeling, drug screening as well as personalized medicine application.

## 4. Conclusions

This study demonstrates the metabolic turnover of glucose and lactate in hiPSC-derived hiAstrocytes as a scalable alternative astrocyte source to HFAs. Using a commercial, multiplexed flow-based biosensor, we were able to demonstrate that hiAstrocytes are as glycogenic as their fetal counterparts, but their normalized metabolic turnover is lower. Our study shows the utility of both hiAstrocytes and flow-based biosensor for metabolic studies. According to our findings, the metabolic enzyme expression is largely within 1 DDCt between the cell types, indicating a comparable intrinsic metabolic capacity, although we find some variation in certain transport genes. Under the culture conditions of the defined media in this study, HFAs showed 2.3-fold higher levels of lactate production compared to hiAstrocytes. In terms of glucose consumption, we observed 2.1-fold higher rates in HFAs than in hiAstrocytes. Despite the differences found in metabolic rates for HFAs and hiAstrocytes, the glycogenic properties that we found in the hiAstrocytes suggest that they can still be used as a relevant cell model for several purposes. The similarity in metabolic enzyme expression level encourages future work for further optimization of differentiation protocols such as the one used in this work. Since HFAs are difficult to access in large amounts and are burdened with ethical considerations, the use of hiPSC-derived astrocytes can bring forward drug screening research, disease modeling, and personalized medicine including metabolic studies starting with a simple skin biopsy.

Last but not least, the metabolic assessment with the commercial, off-the-shelf biosensor proved successful overall. For lactate, we found its use at least on par with, if not superior to, a standard lactate assay (for which we found high variability). By being able to process complex cell culture media samples directly, the sensor eliminates potentially significant sources of error in terms of multiple pipetting, mixing, and incubation steps that feature in traditional plate-based assays. However, care should be taken to frequently measure standard samples to control and compensate for sensor drift. The cost of the utilized biosensor in this study, with a lifespan sufficient for at least ~200 assays based on our present study, is USD 270 per sensor. This equates to only 30% of the per-assay cost compared to typical glucose or lactate assay kits (e.g., USD 120 for 20 assays, and USD 500 for 100 assays, respectively, at Sigma-Aldrich). Therefore, the employed sensor in this study can provide a reproducible, fast, and cost-effective option for detecting glucose and lactate. With its flow-through format, the biosensor approach is especially well-suited for future integration with microphysiological models (organs-on-chips). These can pave the way to achieving more realistic and accurate models for in vitro investigation of metabolic disorders and dietary interventions such as ketone bodies for treating CNS-related diseases such as epilepsy or inborn error of metabolism.

## Figures and Tables

**Table 1:** Metabolic enzymes and transporters considered for qPCR analysis.

Metabolic Enzymes	
BDH2	3-Hydroxybuturate dehydrogenase 2, initiates ketolysis
ABAT	4-Aminobutyrate aminotransferase, catabolizes GABA into succinic semialdehyde.
HMGCL	3-Hydroxy-3-methylglutaryl-CoA lyase, catalyzes the formation of acetoacetate from HMG-CoA
OXCT1	3-Oxoacid CoA-Transferase 1, catalyzes CoA from succinyl-CoA to acetoacetate
**Transporters**	
SLC6A1	Solute carrier family 6 member 1, GABA transporter, GABA reuptake in presynaptic neurons and astrocytes
SLC16A1	Solute carrier family 16 member 1, Monocarboxylate transporter, suggested to transport lactate in astrocytes

## Data Availability

Data is available upon request.

## Supplementary Materials

The following supporting information can be downloaded at: https://www.mdpi.com/article/10.3390/bios12100839/s1, Figure S1: Comparison of mRNA expression levels of house-keeping gene (GAPDH) in hiAstrocytes to HFA. Figure S2: Lactate production from hiPSC-derived astrocytes and HFA, measured using a standard assay kit, with media sampled after 6 h and 24 h. Data is normalized to dsDNA content and cell number. The general trends observed here agree with those from the biosensor.
