# Supplementary material for: Metabolic Assessment of Human Induced Pluripotent Stem Cells-Derived Astrocytes and Fetal Primary Astrocytes: Lactate and Glucose Turnover
Source: Biosensors (Basel). 2022 Oct 8;12(10):839. doi: 10.3390/bios12100839 (PMC9599592; doi:10.3390/bios12100839)
Supplement: Supplementary file 1 [file biosensors-12-00839-s001.zip › biosensors-1896167-supplementary.pdf]

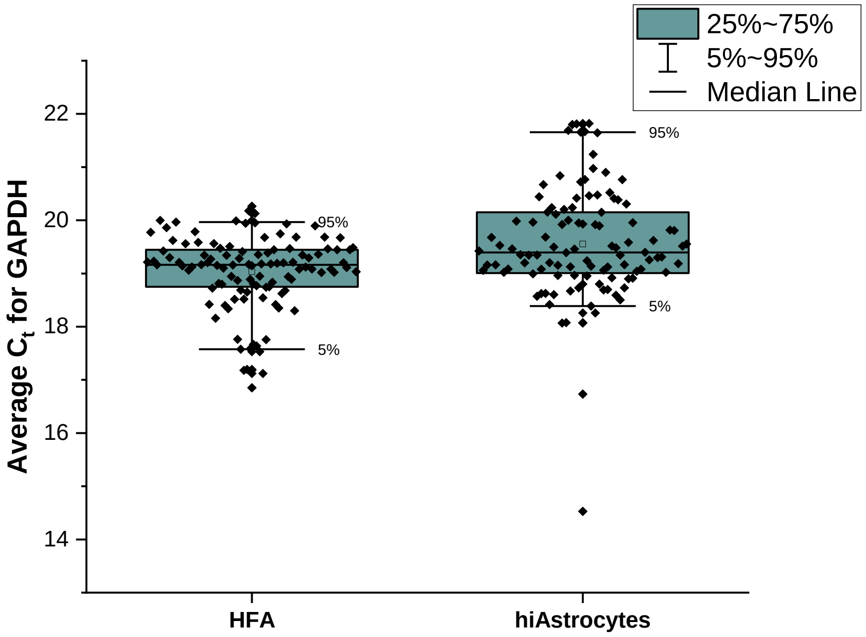

Figure S1: Comparison of mRNA expression levels of house-keeping gene (GAPDH) in hiAstrocytes to HFA.

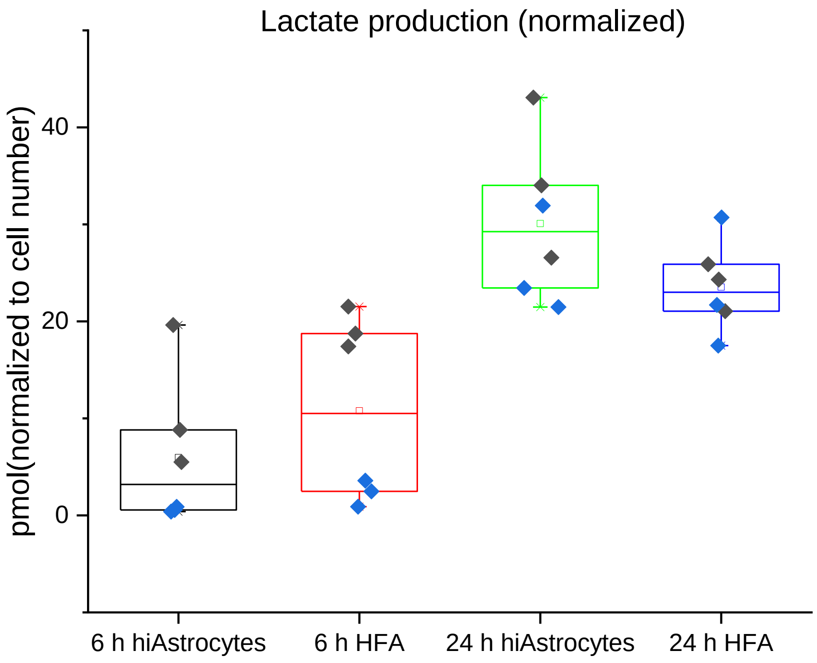

Figure S2: Lactate production from hiPSC-derived astrocytes and HFA, measured using a standard assay kit, with media sampled after 6 h and 24 h. Data are

presented both as a box indicating the 25th–75th percentile, including a median line and ±1.5 IQR whiskers, with the addition of individual data points where colors represent independent experimental rounds. Data is normalized to dsDNA content. The general trends observed here agree with those from the biosensor.
